# OsVPE3 Mediates GA-induced Programmed Cell Death in Rice Aleurone Layers via Interacting with Actin Microfilaments

**DOI:** 10.1186/s12284-020-00376-6

**Published:** 2020-03-30

**Authors:** Heting Zhang, Yu Xiao, Xiaojiang Deng, Hongyu Feng, Zhe Li, Lulu Zhang, Huiping Chen

**Affiliations:** Key Laboratory of Tropical Biological Resources of Ministry of Education, Hainan University, Haikou, 570228 China

**Keywords:** Actin filaments (AFs), Caspase-1, Caspase-3, OsVPE3, Programmed cell death (PCD), Rice aleurone layers

## Abstract

**Background:**

Vacuolar processing enzymes (VPEs) have been identified as the enzymes that regulate vacuole-mediated programmed cell death (PCD) in plants. The mechanism that VPE regulates the PCD in rice aleurone layers remains unknown.

**Results:**

The aleurone layers treated with distilled water exerted caspase-1 and VPE activity, both of which were inhibited by the caspase-1 specific inhibitor Ac-YVAD-CMK but not by the caspase-3 specific inhibitor Ac-DEVD-CHO. However, the caspase-1 and caspase-3 inhibitors weakened the activity of caspase-3. Combined with the effects of endogenous gibberellin (GA) on the induction of OsVPEs, we suggest that the OsVPE3 in the aleurone layers, which exhibits caspase-1-like activity, is a key molecule in GA-induced PCD via regulating the protease with caspase-3-like activity. Many studies have confirmed that vacuolar fusion is an important feature of vacuole-mediated PCD in plants. In this experiment, the process of vacuole fusion was accompanied by changes in the structure of actin filaments (AFs), specifically, their depolymerization and polymerization. The process of vacuolar fusion was accelerated or delayed by the promotion or inhibition of the depolymerization of AFs, respectively. Here, the inhibition of OsVPE3 blocked the depolymerization of AFs and delayed the fusion of vacuoles, indicating that OsVPE3 can regulate the fusion of vacuoles in rice aleurone layers via mediating AFs. Furthermore, the depolymerization of AFs contributed to the up-regulation of *OsVPE3* gene expression and VPE activity, resulting in accelerated PCD in rice aleurone layers. However, the inhibitor of VPE reversed the effects of AF depolymerization on the activity of VPE, then postponing the process of PCD, implying that AF can involve in GA-induced PCD of rice aleurone layers by mediating OsVPE3.

**Conclusions:**

Together, activation of OsVPE3 and depolymerization of AFs shortened the process of vacuolation and PCD in rice aleurone layers, and OsVPE3 interacted with AFs during regulation.

## Background

The programmed cell death (PCD) of aleurone layers is essential for the germination of cereal seeds (Domínguez and Cejudo, 2014). The vacuoles fuse and form a large central vacuole in the aleurone layers, then the large central vacuole ruptures, resulting in the occurrence of PCD (Zheng et al., 2017). Vacuolar processing enzymes (VPEs), which belong to a family of cysteine proteases, are key factors that trigger PCD in plants (Hatsugai et al., 2006; Hatsugai et al., 2015). Previous experiments revealed that activated VPE induces rupture of the tonoplast, which promotes the process of PCD via processing protein kinases in the vacuoles (Sanmartín et al., 2005). Caspases are well-known cysteine proteases with aspartate specificity that play an important role in apoptosis in animals (Woltering 2004; Kumar 2007). Interestingly, VPE is similar in structure and characteristics to caspase-1, particularly because it contains aspartic acid (Asp) pockets that include three key amino acids (Arg-179, Arg-341 and Ser-347) (Hatsugai et al., 2004; Yamada et al., 2005). Studies have shown that VPE exerts caspase-1-like activity in the vacuole-induced cell death of TMV (tobacco mosaic virus) in *Nicotiana* (Hatsugai et al., 2004), and that Ac-ESEN-CHO, a specific inhibitor of VPE, effectively inhibits the occurrence of PCD (Hatsugai et al., 2009; Li et al., 2012). Therefore, it is presumed that VPE in plants has similar activity to caspase-1 in animals. Furthermore, pharmacology experiments showed that specific inhibitors of caspase-1 and caspase-3, Ac-YVAD-CHO and Ac-DEVD-CHO, prevent the DNA strand breaks and the degradation of PARP, thus postponing the occurrence of menadione-induced PCD in tobacco protoplasts (Sun et al., 1999). Both Ac-YVAD-CMK and Ac-DEVD-CHO have already been shown to inhibit PCD of rice aleurone layers at 100 *μ*M (Zheng et al., 2017). Other studies also clarified that inhibition of *OsVPE3* expression reduces the rupture of vacuoles, leading to improved tolerance of rice stomata to salt in the development (Lu et al., 2016).

Actin microfilaments (MFs), also known as actin filaments (AFs), exist in eukaryotic cells in two forms: spherical actin (G-actin) monomers and fibrous actin (F-actin) polymers. AFs regulate several key cellular functions, including cell division, cell elongation, stomatal movement, material transport, cytoplasmic circulation, and signal transduction (Kost and Chua, 2002; Kost et al., 2002; Smith 2003; Wasteneys and Galway, 2003; Kaštier et al., 2018). The dynamic change of AFs between depolymerization and polymerization is necessary for initiating these functions; however, the corresponding physiological functions are executed only in the polymerization state (Kaštier et al., 2018). Cytochalasin D (CD), a depolymerizer of F-actin, inhibits the dynamic change of the vacuole membrane in epidermal cells in *Arabidopsis* leaf (Uemura et al., 2002). Furthermore, cytochalasin B (CB) promoted the process of vacuolation in rice aleurone cells, whereas the stabilizer phalloidin effectively inhibited the process (Zheng et al., 2017). Therefore, it is speculated that AF is involved in regulating the dynamic changes of vacuoles. This hypothesis is supported by experiments which indicated that the rearrangement of AF is related to the dynamic changes of vacuoles in plants (Uemura et al., 2002) and the fusion of vacuoles in yeast (Eitzen et al., 2002). A dynamic wave structure appears on the surface of the vacuoles in the protoplasts of tobacco, and the structure disappears in the CB-treated protoplasts but not those treated with the microtubule depolymerizer oryzalin (Verbelen and Tao, 1998). This illustrates that the dynamic wave structure of the vacuole is mainly regulated by AF. Moreover, an F-actin depolymerizer inhibited the occurrence of PCD in the embryos of *Picea abies* (Smertenko et al., 2003), implying that the integrity of the AF structure plays a key role in PCD during the normal development of plants.

Our previous study showed that the CB and a caspase-1 inhibitor effectively promoted or delayed PCD in rice aleurone layers (Zheng et al., 2017). Based on this observation, we further explored the mechanism of VPE in mediating PCD in rice aleurone layers via interaction with AFs. Our results revealed that endogenous GA-induced OsVPE3 has caspase-1-like activity and is required for PCD in the aleurone layers during the germination of rice seeds. Our results also showed that the morphology of vacuoles was influenced by the change of AF structure. Moreover, the AF depolymerizer not only up-regulated the activity of VPE, but also accelerated the vacuolation of vacuoles, resulting in promoting the PCD of aleruone layers. Consistent with the inhibition of VPE activity, vacuolation was blocked by the inhibitor Ac-YVAD-CMK and aleurone PCD was postponed. Importantly, Ac-YVAD-CMK reversed the promotion effect of the AF depolymerizer. These findings reveal the mechanisms of OsVPE3-mediated initiation of PCD in rice aleurone layers.

## Results

### The *OsVPE3* mRNA of Aleurone Layers Changes in a Time-dependent Manner at the Early Stage of Culture

**Fig. 1:**
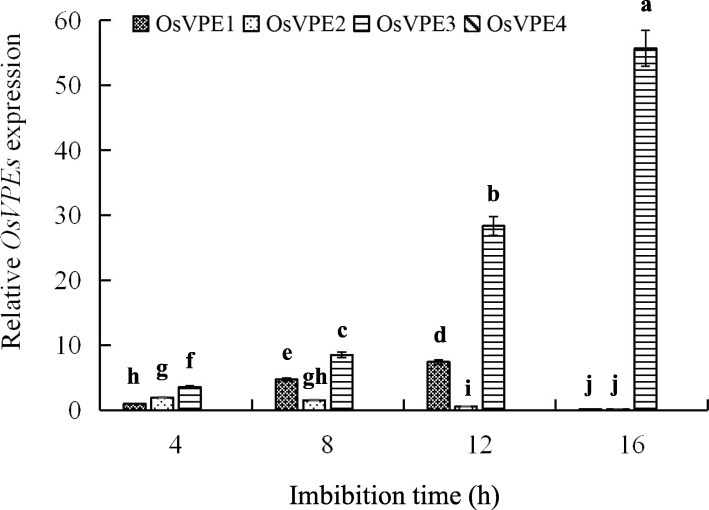
The levels of *OsVPE3* gene expression changed over time. Aleurone layers were stripped from the intact seeds imbibed for 4, 8, 12, and 16 h, and then the mRNA transcripts of *OsVPE1*, *OsVPE2*, *OsVPE3*, and *OsVPE4* were measured. The expression of *OsVPE1* in 4 h was selected as 1. Error bars represent the means ± s.d., *n* = 3 biological independent experiments with at least three replicates for each. Bars with different alphabets show significant different at *P*<0.05 by Duncan’s multiple range test

The four VPE genes, *VPE1*, *VPE2*, *VPE3*, and *VPE4*, were identified in rice (Wang et al., 2009). Furthermore, the four VPE are related to the germination of rice seeds (Deng et al., 2011). Thus, to identify the key VPE regulating the germination of rice seeds in the aleurone layers, we measured the transcription levels of the four VPE genes in the aleurone layers isolated from the intact seeds cultured in distilled water for 4, 8, 12, and 16 h. As shown in Fig. 1, the transcription level of *OsVPE3* significantly increased from 4 to 16 h, while that of *OsVPE1* significantly decreased. Similar to that of *OsVPE3*, the expression level of *OsVPE2* rose from 4 to 12 h, but sharply declined at 16 h. Moreover, the transcription level of *OsVPE4* was not detected. Importantly, the transcription levels of *OsVPE3*, especially at 12 and 16 h, were significantly higher than those of *OsVPE1*, and *OsVPE2* in aleurone layers. This indicated that OsVPE3 in aleurone layers is a major contributor to the germination of rice seeds.

### OsVPE3 is Mainly Regulated by Endogenous GA in the Aleurone Layers of Rice

**Fig. 2:**
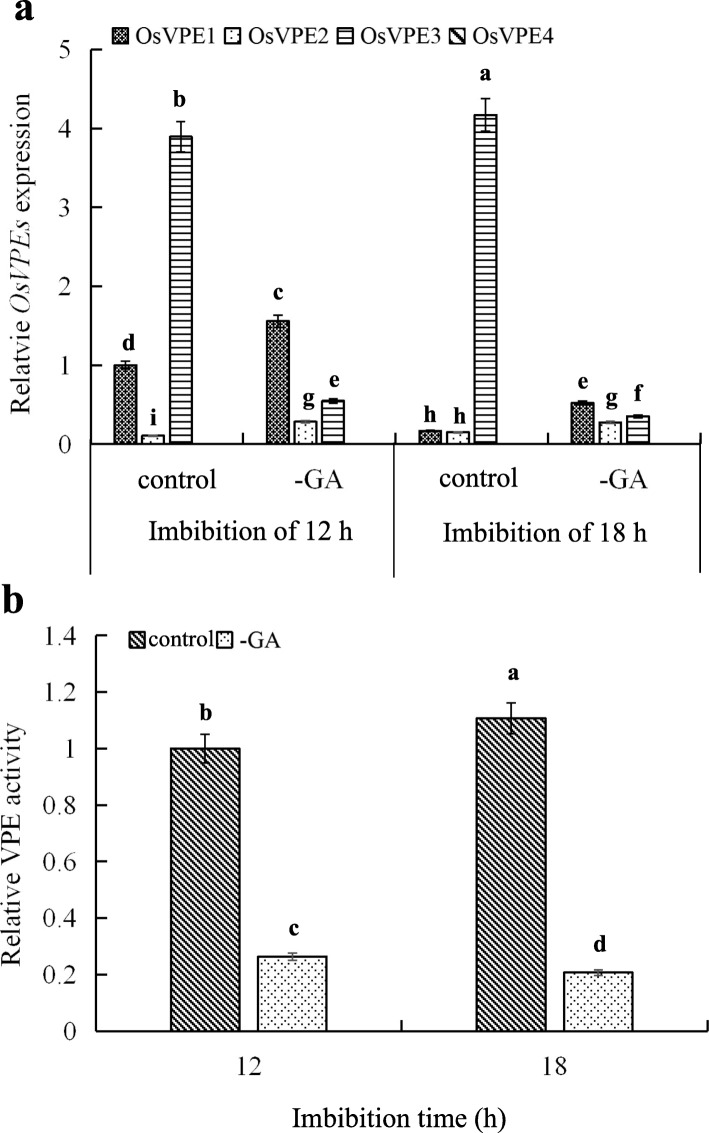
GA raised the levels of *OsVPE3* mRNA transcript expression and VPE activity. The relative levels of *OsVPE1-4* expression (**a**) and VPE activity (**b**) in the aleurone layers obtained from intact seeds (control) and de-embryo seeds (GA) after 12 h and 18 h of imbibition. The *OsVPE1* expression (**a**) and VPE activity (**b**) in the aleurone layers isolated from the intact seeds imbibed for 12 h were selected as 1. Error bars represent the means ± s.d., *n* = 3 biological independent experiments with at least three replicates for each. Bars with different alphabets show significant different at *P*<0.05 by Duncan’s multiple range test

Exogenous GA is involved in advancing the PCD process of aleurone layers in rice (Zhang et al., 2018; Wu et al., 2016), barley (Bethke et al., 1999) and wheat (Bissenbaev et al., 2011; Xie et al., 2014), contributing to the germination of cereal seeds. To verify whether endogenous GA regulates the OsVPE3 of aleurone layers of germinating rice seeds, we measured the transcription levels of *OsVPE1*, *OsVPE2*, *OsVPE3*, and *OsVPE4* and the activity levels of VPE in the aleurone layers of intact seeds and de-embryo (-GA) seeds (Fig. 2). Compared with de-embryo seeds, the *OsVPE3* gene expression of aleurone layers in intact seeds significantly increased, whereas that of *OsVPE1* significantly decreased, and that of *OsVPE2* was a slightly increase. In the aleurone layers of the intact seeds imbibed in distilled water from 12 to 18 h, the transcript level of *OsVPE3* obviously increased, while that of *OsVPE1* obviously fell. In contrast, in the aleurone layers of the de-embryo seeds imbibed in distilled water from 12 to 18 h, the transcription levels of *OsVPE1* and *OsVPE3* decreased, while that of *OsVPE2* remained unchanged. In addition, no gene expression of *OsVPE4* was measured in the aleurone layers of intact seeds and de-embryo seeds (Fig. 2a). These results suggesting that endogenous GA is secreted from embryos to aleurone layers, and then induces the expression of *OsVPE3*. Furthermore, the levels of VPE activity in the aleurone layers of intact seeds significantly rose, whereas decreased in the aleurone layers of de-embryo seeds (Fig. 2b). Based on the above results, it is speculated that the increased activity of VPE in the aleurone layers of intact seeds may be mainly up-regulated by *OsVPE3*. Therefore, a conclusion can be drawn that endogenous GA regulates OsVPE3 in the aleurone layers of germinating rice seeds.

### OsVPE3 Exhibits Specific Caspase-1-like Activity in Rice Aleurone Layers

**Fig. 3:**
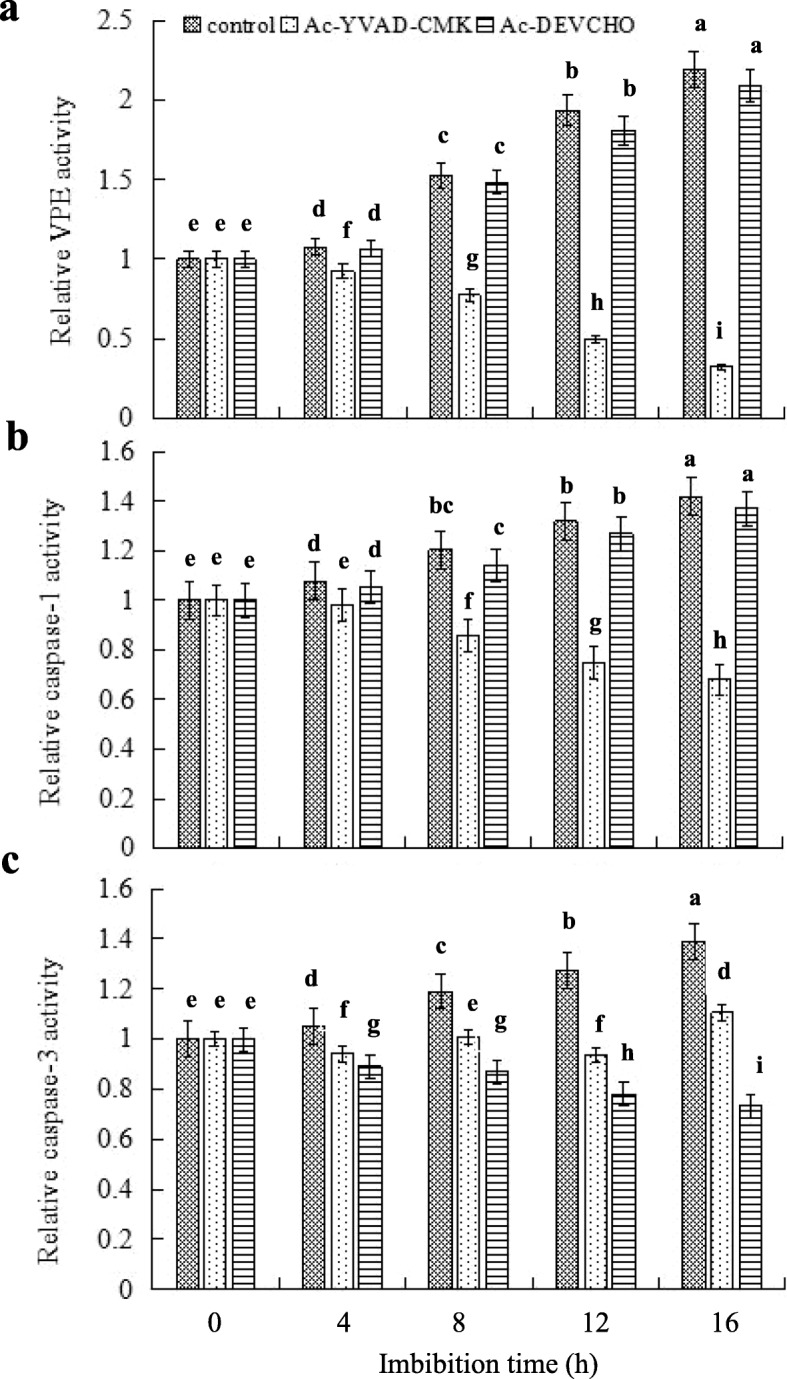
OsVPE exhibited caspase-1-like enzymatic activity. Aleurone layers were stripped from the intact seeds imbibed for 12 h, and then the isolated layers were pre-incubated in a solution containing distilled water (control) alone or 10 *μ*M Ac-YVAD-CMK (caspase-1 inhibitor), and 10 *μ*M Ac-DEVD-CHO (caspase-3 inhibitor) alone or a combination for 0, 4, 8, 12, and 16 h. Then, the activities of VPE (**a**), caspase-1 (**b**), and caspase-3 (**c**) were measured. Error bars represent the means ± s.d., *n* = 3 biological independent experiments with at least three replicates for each. Bars with different alphabets show significant different at *P*<0.05 by Duncan’s multiple range test

To analyze the protease property of VPE, we examined the activities of VPE, caspase-1- and caspase-3-like in aleurone layers treated with the inhibitors of caspase-1 and caspase-3. As shown in Fig. 3, VPE activity exhibited similar changes in the controls (treated with distilled water) and the layers treated with the caspase-3 specific inhibitor Ac-DEVD-CHO. This implies that caspase-3 inhibitor had no effect on the activity of VPE. However, the caspase-1 specific inhibitor Ac-YVAD-CMK, significantly inhibited VPE activity, and the inhibitory effect was more obvious with prolonged treatment time (Fig. 3a). The change in caspase-1 activity was consistent with that of VPE activity in the aleurone layers treated with distilled water, while the effect of the caspase-1 inhibitor on caspase-1 activity was also consistent with that of VPE (Fig. 3b). Thus, it can be concluded that VPE has activity similar to caspase-1 but does not have caspase-3-like activity (Kuroyanagi et al., 2005). Furthermore, caspase-3 activity in aleurone layers treated with distilled water showed an upward trend which was significantly suppressed by Ac-DEVD-CHO and Ac-YVAD-CMK (Fig. 3c). Accordingly, the inhibitory activity of the latter was less than that of the former. It appears that the protease with caspase-1-like activity mediated by the protease with caspase-3-like activity plays an important role in the aleurone layers of rice. In short, the above results suggest that VPE in rice aleurone layers has caspase-1-like activity but not caspase-3-like activity.

### The Depolymerization of AFs Contributes to the Levels of *OsVPEs* Gene Expression and VPE Activity in Rice Aleurone Layers

**Fig. 4:**
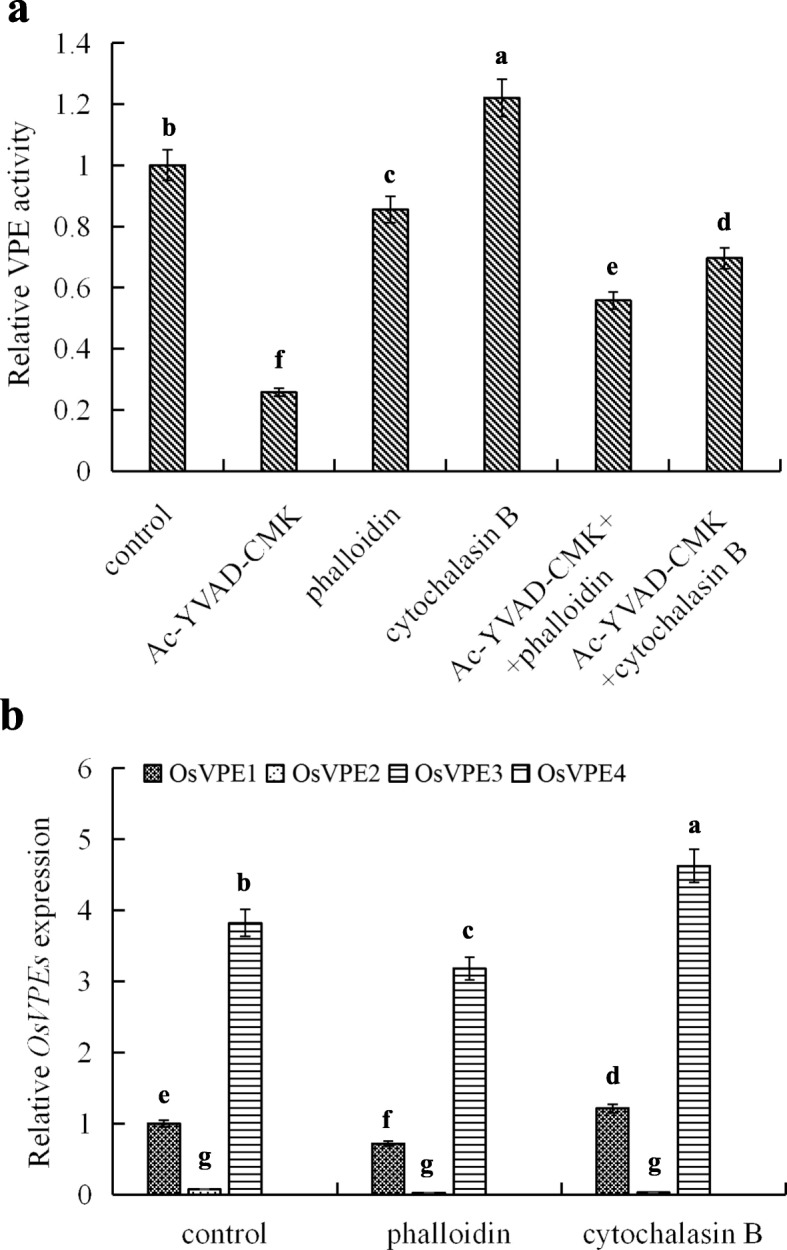
The depolymerization of AF increased the levels of *OsVPE3* mRNA transcription and VPE activity. Aleurone layers were stripped from the intact seeds imbibed for 12 h, and then the isolated layers were pre-incubated in a solution containing distilled water alone (control) or 10 *μ*g/mL phalloidin (AF depolymerization inhibitor), 10 *μ*g/mL cytochalasin B (CB) (AF depolymerizer), 10 *μ*M Ac-YVAD-CMK (caspase-1 inhibitor) alone or in combination for 12 h. VPE activities (**a**) and *OsVPE1-4* mRNA transcripts (**b**) were measured. The VPE activity and *OsVPE1* expression in the control were selected as 1. Error bars represent the means ± s.d., *n* = 3 biological independent experiments with at least three replicates for each. Bars with different alphabets show significant different at *P*<0.05 by Duncan’s multiple range test

To investigate the impact of AFs on the levels of *OsVPEs* gene expression and VPE activity, we treated aleurone layers with the AF depolymerizer cytochalasin B (CB) and the stabilizer (or depolymerization inhibitor) phalloidin. Compared with the control, the activity level of VPE in the aleurone layer treated with CB increased by 22.1% (*P*<0.05), while the activity of VPE significantly decreased by 14.5% (*P*<0.05) and 74.3% (*P*<0.05) in the aleurone layers treated with phalloidin alone and Ac-YVAD-CMK alone, respectively (Fig. 4a). However, the combined CB and Ac-YVAD-CMK treatment inhibited the VPE activity of layers 43.0% (*P*<0.05) compared to CB treatment alone, and the treatment of phalloidin+Ac-YVAD-CMK hindered the activity by 29.7% (*P*<0.05) compared to phalloidin treatment alone (Fig. 4a). This result indicates that the AF depolymerizer and stabilizer significantly up-regulates or down-regulates VPE activity. Ac-YVAD-CMK effectively blocked AF depolymerizer-induced up-regulation of VPE activity and effectively promoted the AF stabilizer-induced down-regulation of VPE activity. Therefore, to some extent, the depolymerization of AFs activated VPE activity in aleurone layers, and thus it is speculated that VPE activity is regulated by AF.

The changes in *OsVPEs* expression were consistent with that of VPE activity in the aleurone layers treated with AF depolymerizer alone or AF stabilizer alone. As shown in Fig. 4b, the treatment of CB alone raised the transcription levels of *OsVPE1* and *OsVPE3*, which were 21.4% and 21.0% (*P*<0.05) higher than that of the distilled water treatment, respectively. In contrast, the treatment of phalloidin alone significantly decreased the gene expression levels of *OsVPE1* and *OsVPE3*, which were 28.3% and 20.2% (*P*<0.05) lower than that of distilled water treatment, respectively. Moreover, the gene expression levels of *OsVPE3* were much higher than that of *OsVPE1* in the treatment of distilled water or phalloidin alone, CB alone. However, the transcription level of *OsVPE2* was not affected by the treatment of AF depolymerizer alone or AF stabilizer alone, and no transcription of *OsVPE4* was detected in all treatments. Combined with the above experimental results, we concluded that AFs regulate the transcription levels of *OsVPE3* and VPE activity.

### Different Vacuolar Morphology Corresponds to Different AF Characteristics in the Aleurone Cells of Rice

**Fig. 5:**
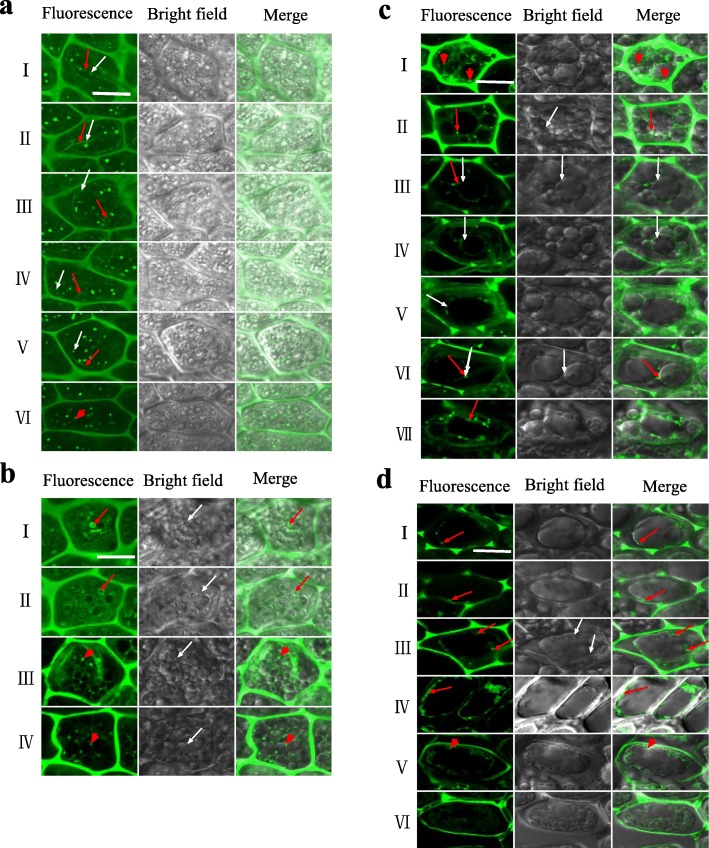
The changes of AF structure influenced vacuolar morphology. Aleurone layers were stripped from the intact seeds imbibed for 12 h, and then the isolated layers were pre-incubated in distilled water alone from 1 d to 7 d, the incubated layers were labeled with FITC-phalloidin, the organization of AF (green) was observed by LSCM, and images were immediately captured. The experiments were repeated three times, and similar images were obtained. Scale bar is 10 *μ*m. **a** The tiny vacuoles and the morphological changes of AFs. **b** The small vacuoles and the morphological changes of AFs. **c** The larger vacuoles and the morphological changes of AFs. **d** The large central vacuoles, the shrinking protoplast and the morphological changes of AFs

Our previous morphological experiment demonstrated that the AF depolymerizer and stabilizer promotes or hinders the coalescence of vacuoles, thereby accelerating or delaying the PCD of rice aleurone layers (Zheng et al., 2017). However, we have not observed the changes of AF structure using laser scanning confocal microscopy (LSCM) during the process of vacuolation. Here, to investigate the correlation between the change of AF structure and the morphology of vacuoles, we labeled AFs using a FITC-labeled phalloidin fluorescent probe, and then observed the morphological characteristics of AFs and vacuoles under LSCM. When the aleurone cells were filled with tiny vacuoles, a green, irregular, and dispersed filamentous structure (Fig. 5a, I-V; red arrow) and a tiny granular structures appeared (Fig. 5a, I-V; white arrow) simultaneously. When tiny vacuoles fused to slightly larger vacuoles, the filamentous structure disappeared while AF with a short columnar structure appeared (Fig. 5a, VI; red arrow head), indicating that AF does not depolymerize on the tiny vacuole.

When the slightly larger vacuoles occurred in the cell, the AF surrounded the smaller vacuoles (Fig. 5b, I-II; red arrow). In addition, the AF with a short columnar structure appeared in the location close to where the membranes of two vacuoles were in contact with each other (Fig. 5b, III-IV; white arrow), suggesting that the filaments of AF polymerize to a ring-like structure and then to a short columnar structure (Fig. 5b, III-IV; red arrow head). Following the change of AF structure, the smaller vacuoles gradually fused to slightly larger vacuoles in the aleurone cells.

When one or more larger vacuole appeared in a cell, the AFs with a short columnar structure surrounded them (Fig. 5c, I; red arrow head). However, the membrane of the larger vacuole was in close contact with the membrane of another vacuole (Fig. 5c, II-VI; white arrow), and the short columnar structure of AFs did not appear at the membrane fusion site, whereas the structure emerged around the fusion site of the membranes (Fig. 5c, II-III; red arrow). On the tonoplast, where the two larger vacuoles were close to each other without touch (Fig. 5c, VI; white arrow), the short columnar structure of AFs appeared (Fig. 5c, VI; red arrow). When the two larger vacuoles coalesced through membrane fusion, the fusion body of the two vacuoles was long, i.e. the fusion had not completed, and the short columnar structure of AFs was also observed on the elongated vacuoles (Fig. 5c, VII; red arrow). These results revealed that the AFs do not depolymerize on the tonoplast before the coalescence of vacuoles occurs. In addition, when the membranes of two vacuoles contact with each other, the AF at the contact site had depolymerized, and when the vacuole fusion began, the AF repolymerized.

When larger vacuoles fused to a large central vacuole, the short columnar structure of AFs was observed on the membrane of the large central vacuole (Fig. 5d, I; red arrow). When the large central vacuole elongated and deformed, the short columnar structure was longer than that on the large central vacuole and longitudinally attached to the tonoplast (Fig. 5d, II; red arrow). It appears that the extension of the large central vacuole was due to the dynamic change of AF. When the large central vacuole ruptured (Fig. 5d, III; white arrow), the short columnar structure appeared at the rupture site with a slightly higher fluorescence intensity (Fig. 5d, III; red arrow), implying that the burst of vacuoles also requires the dynamic change of AF. The rupture of the large central vacuole led to the collapse of the plasma membrane, and the disintegrated vacuole and plasma membrane mixed together, while many short columnar structures appeared at the junction of the protoplast and the cell wall (Fig. 5d, VI; red arrow). When the protoplast began to contract inward without obvious boundaries between it and the cell wall, the short columnar structure of AFs was observed on the protoplast (Fig. 5d, V; red arrow head). Then, the protoplast was distinctly separated from the cell wall in some areas and the short columnar structure did not appear. The whole mass of the protoplast contracted inward, resulting in a clear boundary between it and the cell wall. At this time, the short columnar structure was not observed and only green dispersion remained on the protoplast (Fig. 5d, VI), indicating that AFs had depolymerized and even actin had begun to dissolve. Therefore, our results suggested that the regulation of AF structure is crucial for maintaining vacuolar morphology.

### OsVPE3 Contributes to the Depolymerization of AFs in the Process of Vacuolation in the Aleurone Cells of Rice

**Fig. 6:**
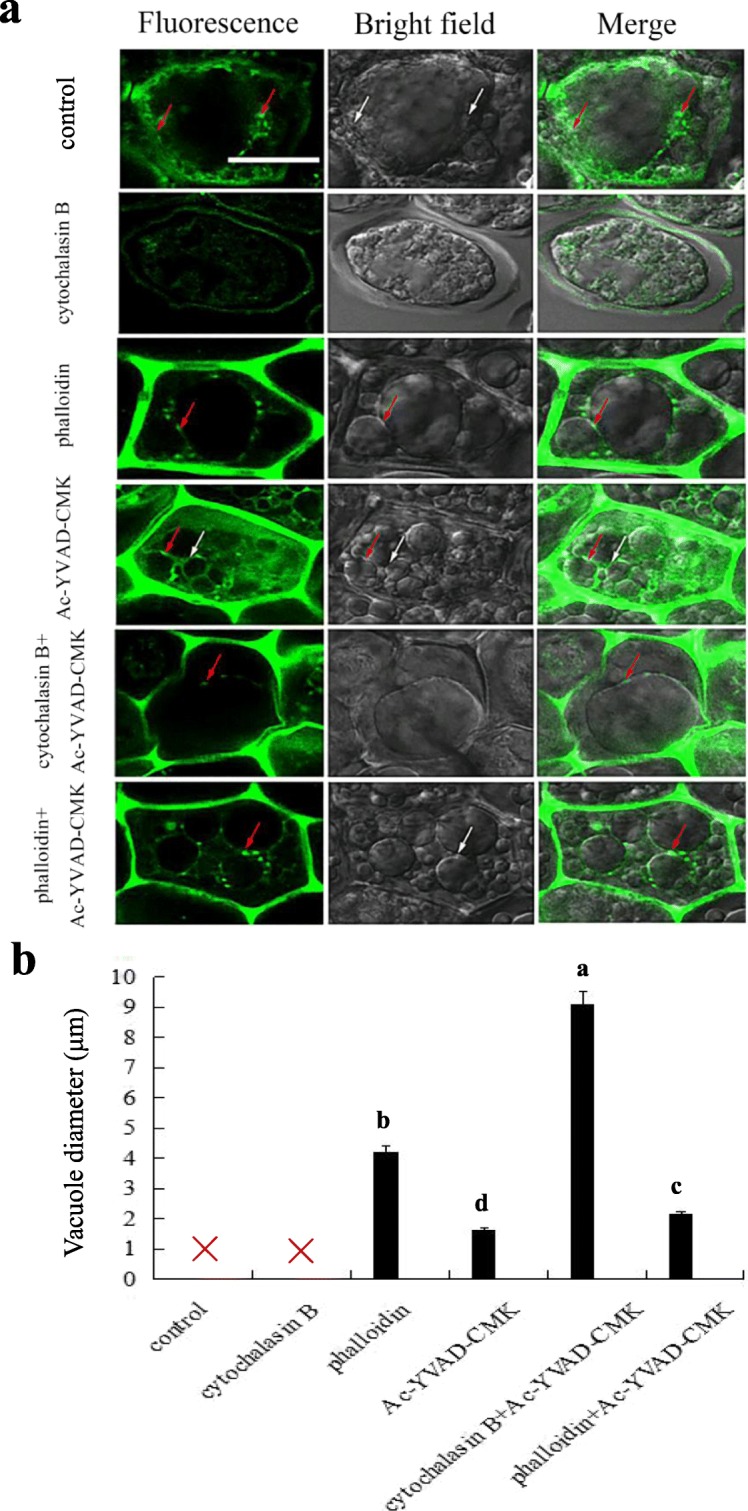
Depolymerization blocked the process of vacuole fusion. Aleurone layers were stripped from the intact seeds imbibed for 12 h, and then the isolated layers were incubated in a solution containing distilled water (control) alone or 10 *μ*g/mL phalloidin (AF depolymerization inhibitor), 10 *μ*g/mL cytochalasin B (CB) (AF depolymerizer), 10 *μ*M Ac-YVAD-CMK (caspase-1 inhibitor) alone or in combination for 7 d, the layers were labeled with FITC-phalloidin, **a** the organization of AF (green) was observed by LSCM, and images were immediately captured. Scale bar is 10 *μ*m. **b** Statistical analyses were conduced on the vacuole diameter per cell. Red cross represents the ruptured large central vacuole. Error bars indicate the means ± s.d., *n* = 3 biological independent experiments with at least three replicates for each. Bars with different alphabets show significant different at *P*<0.05 according to Duncan’s multiple range test

To further understand the effects of AF deploymerizaiton and polymerization on the process of vacuolation, we observed the AF structute and vacuole morphology in the aleurone cells treated with distilled water, CB, phalloidin, and Ac-YVAD-CMK alone or combination for 7 d. As shown in Fig. 6a, the short columnar structure of AF (red arrow) was observed in the uneven part of the vacuole in the untreated control, and this was the rupture site of the large central vacuole (white arrow). Simultaneously, the protoplast contracted and was covered with the green dispersion in the cells of aleurone layers treated with CB alone, indicating that the AF had disintegrated. Both the larger and the slightly larger vacuoles were close to each other in the cells of aleurone layers treated with AF stabilizer phalloidin alone, and the short columnar structure was observed at the close contact site of vacuoles with a average diameter of 4.19 *μ*m (Fig. 6b). These results suggested that the vacuolation process of aleurone cells treated with AF depolymerizer was faster than that in the distilled water treatment, while that treated with AF stabilizer was much slower. Consequently, we deduced that the depolymerization of AF contributes to the fusion of vacuoles, while the inhibition of AF depolymerization hinders the fusion of vacuoles.

To determine the relationship between AF and VPE in aleurone cells, we further observed the structure of AF in the cells of Ac-YVAD-CMK-treated aleurone layers (Fig. 6). We found that the slightly larger vacuoles with the average diameter of 1.63 *μ*m appeared in the aleurone cells treated with Ac-YVAD-CMK alone, and the volume was smaller than that in the distilled water treatment, implying that the vacuoles were still at the stage of fusion (white arrow). The short columnar structure of the AF appeared around the vacuoles that would fuse (red arrow), suggesting that the inhibition of VPE stops the depolymerization of AF in aleurone cells, postponing the coalescence of vacuoles. Therefore, we deduced that VPE regulates vacuolar fusion through AF.

Furthermore, the co-treatment of CB and Ac-YVAD-CMK effectively retarded the rate of CB-accelerated vacuolar rupture, thus a large central vacuole that the average diameter is 9.07 *μ*m was observed in the cell, and a short columnar structure anchored on the tonoplast (red arrow). The slightly larger vacuoles with a average diameter of 2.14 *μ*m were distributed in the cells of aleurone layers co-treated with phalloidin and Ac-YVAD-CMK, which were smaller than those treated with phalloidin alone. These vacuoles were very close to each other but not in contact with each other, and the short, green columnar structure of AF was located on the membrane of the two slightly larger vacuoles that were very close to each other. This suggests that Ac-YVAD-CMK effectively inhibits the effects of the CB and phalloidin, thus it is assumed that the inhibition of VPE affects the deploymerizaiton and polymerization of AF, and then delays the fusion of vacuoles. Based on the above results, we concluded that OsVPE3 regulates the fusion of vacuoles in the aleurone layers of rice by mediating AF

### OsVPE3 and AF Promote the PCD Process in Aleurone Layers in Rice

**Fig. 7:**
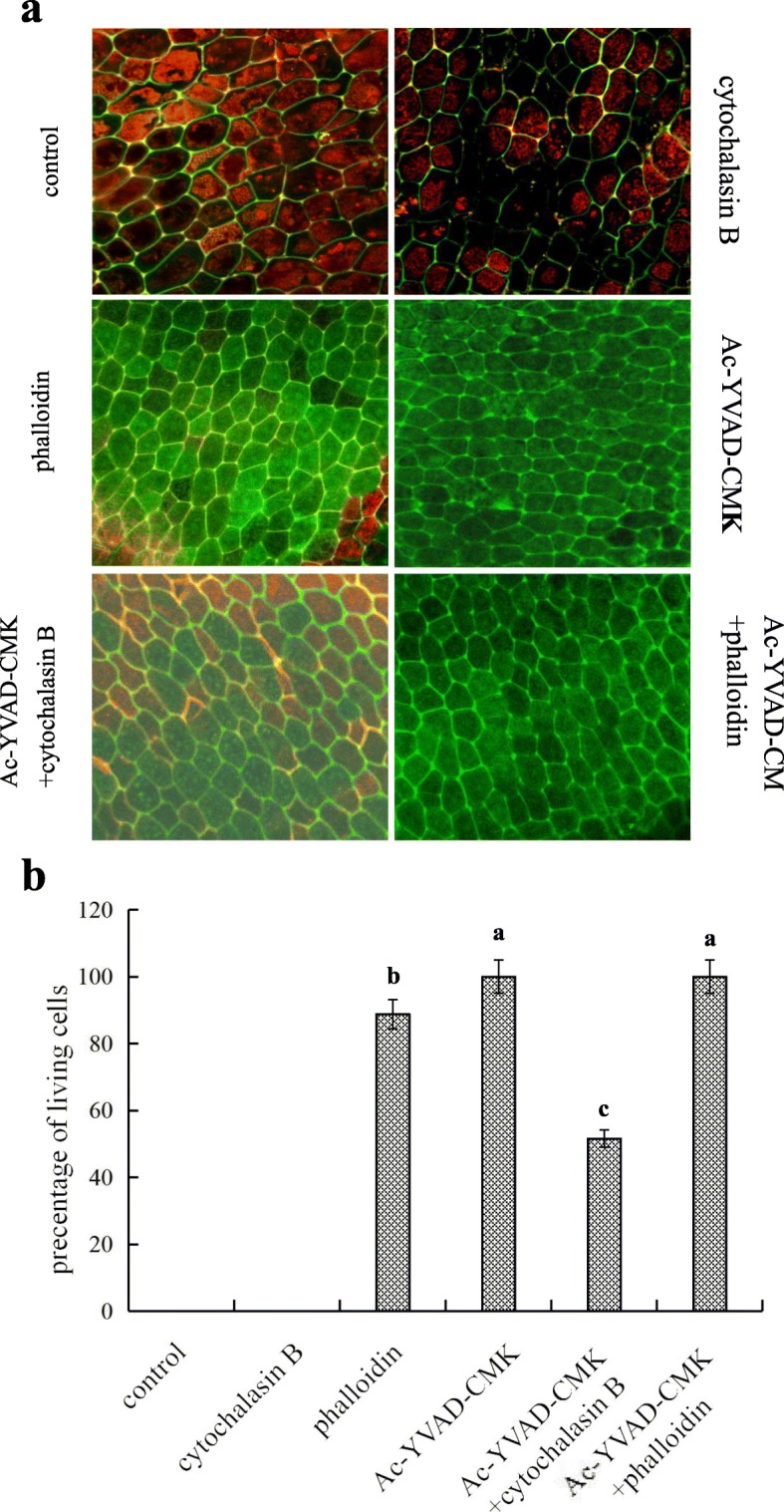
The inhibition of *OsVPE3* delayed the process of PCD in rice aleurone layers. Aleurone layers were stripped from the intact seeds imbibed for 12 h, and then the isolated layers were pre-incubated in a solution containing distilled water alone (control) or 10 *μ*g/mL phalloidin (AF depolymerization inhibitor), 10 *μ*g/mL cytochalasin B (CB) (AF depolymerizer), 10 *μ*M Ac-YVAD-CMK (caspase-1 inhibitor) alone or in combination for 7 d. Finally, the stained layers with FM-4-64 (orange or red, dead cells) and FDA (green, live cells) were observed to distinguish live cells and dead cells under LSCM, and the corresponding images were immediately captured. **a** The captured images were performed three times and a representative aleurone layer section is shown. Scale bar is 10 *μ*m. **b** The survival rate of cells was quantified for at least three aleurone layers. Error bars indicate the means ± s.d., *n* = 3 biological independent experiments with at least three replicates for each. Bars with different alphabets show significant different at *P*<0.05 according to Duncan’s multiple range test

To determine the effects of AF structure change and OsVPE3 on triggering PCD, we analyzed the cell viability of aleurone layers treated with the CB, the phalloidin, and the Ac-YVAD-CMK via the double fluorescence agents FDA and FM4-64. As shown in Fig. 7, some cells in the aleurone layers treated with CB emitted a strong red fluorescence and some cells had a cavity, while the cells treated with distilled water emitted orange red (Fig. 7a). Although the process of cell death in aleurone cells treated with the AF depolymerizer was faster than that in the cells treated with distilled water, both of viability of aleurone cells was 0% (Fig. 7b). In contrast, all the cells in the aleurone layers treated with Ac-YVAD-CMK alone emitted green fluorescence without red fluorescence (Fig. 7a), i.e. the cell survival rate of aleurone layers was 100% (*P*<0.05) (Fig. 7b). Whereas some cells emitted green fluorescence in the aleurone layers treated with CB plus Ac-YVAD-CMK (Fig. 7a), and the survival rate of the cells in the co-treatment of CB and Ac-YVAD-CMK was higher than that of the cells treated with CB alone 51.6% (*P*<0.05) (Fig. 7b), implying that the addition of Ac-YVAD-CMK significantly alleviated the effect of the AF depolymerizer on accelerating cell death. In addition, most of the aleurone cells treated with the AF depolymerization inhibitor emitted green fluorescence, and only a small number of cells emitted red fluorescence (Fig. 7a), correspondly, the cell survival rate of aleurone layers was 88.8% (*P*<0.05) (Fig. 7b). The above results confirmed that the AF depolymerizer and depolymerization inhibitor significantly promoted or inhibited the occurrence of aleurone PCD, whereas the VPE inhibitor significantly weakened the effect of the AF depolymerizer in promoting PCD. In conclusion, the inhibition of VPE activity in rice aleurone layers or the depolymerization of AF effectively delays the process of PCD, while promoting the depolymerization of AF induces the occurrence of PCD.

## Discussion

Previous studies confirmed that VPE is involved in the regulation of the abortion-induced PCD process of floral buds in the radish (Zhang et al., 2013). Moreover, VPE regulated the accumulation of sugar in tomato fruits and the hypersensitivity to pathogenic elicitors in plant leaves (Hatsugai et al., 2004; Ariizumi et al., 2011), suggesting that VPE plays an important role in plant growth and development. Zhang et al. (2018) detected the levels of *OsVPE1-4* gene expression in the intact seeds imbibed for 12 h and in the aleurone layers, embryos, and endosperm of the intact seeds imbibed for 12 h, the results revealed that the transcription level of *OsVPE3* was much higher than these of *OsVPE1*, *OsVPE2*, and *OsVPE4* in the intact seeds, aleurone layers, and embryos. In this study, the expression level of *OsVPE3* in aleurone layers dramatically increased following 4-16 h incubation of intact rice seeds (Fig. 1), indicating that the OsVPE3 of rice aleurone layers changes with the course of germination. Furthermore, endogenous GA significantly up-regulated the levels of *OsVPE3* gene expression and VPE activity in the aleurone layers of the intact seeds imbibed for 12 and 18 h (Fig. 2). Given this, in the following experiments, we stripped aleurone layers from the intact seeds imbibed for 12 h, and then treated the layers with different solutions. This not only easily isolated aleurone layers from rice seeds, but also confirmed that the induction of endogenous GA plays a critical role in this study. Interestingly, previous studies suggested that exogenous GA induced the transcription level of *OsVPE3* in rice aleurone layers (Zhang et al., 2018) and the occurrence of PCD in cereal aleurone layers (Bethke et al., 1999; Fath et al., 2000; Zhang et al., 2018). Wu et al. (2016) also showed that exogenous GA promotes the fusion of vacuoles and accelerates the process of PCD in aleurone layers under drought stress.

When we explored whether VPE had caspase-like activity, we found that Ac-YVAD-CMK, a specific inhibitor of caspase-1, significantly reduced VPE activity in rice aleurone layers, while Ac-DEVD-CHO, a specific inhibitor of caspase-3, did not. In addition, the changing trend of VPE activity was consistent with that of caspase-1 activity in the aleurone layers treated with distilled water, indicating that the VPE of rice aleurone layers has caspase-1-like activity but not caspase-3-like activity. Previous reports also confirmed that StVPE1 is not only specific to the substrate of VPE, but also to the substrate of caspase-1, but not to the substrate of caspase-3 (Teper-Bamnolker et al., 2017). Consistent with this, the VPEs exhibit caspase-1-like activity during PCD induced by heat stress, pathogen infection stress, and aluminum stress (Hatsugai et al., 2004; Li et al., 2012; Kariya et al., 2013). It has been shown that VPE is the only protease with caspase-1-like activity in plant (Cai et al., 2014). Based on the above evidence, the caspase-1 specific inhibitor Ac-YVAD-CMK was used instead of the VPE specific inhibitor in pharmacological experiments to obtain true and credible results. By determining the levels of caspases activity, we found that the caspase-1 specific inhibitor Ac-YVAD-CMK and the caspase-3 specific inhibitor Ac-DEVD-CHO significantly inhibited the activity of caspase-3. Moreover, the inhibitory effect of the caspase-3 specific inhibitor on caspase-3 activity was much higher than that of the caspase-1 specific inhibitor. It is reported that the caspase-1 specific inhibitor Ac-YVAD-CHO significantly alleviates vacuole rupture and delays caspase-3 activation in *Arabidopsis thaliana* during high temperature stress (Li et al., 2012). Thus, we propose that the protease with caspase-1-like activity plays a role in the aleurone layers of rice via mediating the protease with caspase-3-like activity.

Vacuole fusion is an important process in rice aleurone layers during the occurrence of PCD (Zheng et al., 2017). In different systems, the process of tonoplast fusion is accompanied by the structural change of AF including AF stabilization, depolymerization, and repolymerization (Koffer et al., 1990; Jahraus et al., 2001). Interestingly, small vacuoles constantly fused to form the large central vacuoles, and then the vacuoles ruptured, which was accompanied by the changes in AF structure (Fig. 5). Furthermore, in agreement with previous findings that the AF stabilizer (or depolymerization inhibitor) inhibited vacuole fusion (Ayscough 2000; Zheng et al., 2014), our pharmacology experiment revealed that the AF depolymerizer and AF stabilizer effectively promoted or inhibited the depolymerization of AF, accelerating or delaying the process of vacuole fusion in rice aleurone cells (Fig. 6). In previous study, we also found that the AF depolymerizer and stabilizer promotes or hinders the coalescence of vacuoles, but we did not observe the changes of AF structure and vacuole morphology in the process of vacuole fusion (Zheng et al., 2017). Based on the results in this study, we concluded that the start-up of vacuole fusion requires the depolymerization of AF, and the repolymerization of AF is also essential for completing the process of vacuole fusion. Consistent with our conclusion, previous studies confirmed that AFs rapidly depolymerize on the vacuoles in the process of vacuole fusion (Isgandarova et al., 2007), and destroying the structure of AF might promote vacuole division or suppress vacuole fusion (Zheng et al., 2014; Mathur et al., 2003).

Surprisingly, we found that Ac-YVAD-CMK, acting as a caspase-1 inhibitor as well as an inhibitor of VPE, inhibits the activity of VPE and delays the fusion of vacuoles, indicating that OsVPE3 regulates the fusion process of vacuoles by mediating AF. Furthermore, the co-treatment with Ac-YVAD-CMK and the AF depolymerizer CB blocked the effect of CB in promoting the fusion of vacuoles. Conversely, Ac-YVAD-CMK enhanced the effect of the AF stabilizer phalloidin in delaying the fusion of vacuoles. These results demonstrated that the fusion of vacuoles in rice aleurone cells requires the involvement of AF dynamics in regulating structural changes of AF, inferring that the depolymerization and polymerization of AF promotes or inhibits vacuole fusion. It is noteworthy that OsVPE3 mediates the fusion of vacuoles in rice aleurone cells by regulating AF. Previous reports indicated that the dynamic change of AF structure affects the structure of vacuoles and is involved in elicitor-induced PCD in the BY-2 cells of tobacco (Higaki et al., 2007). In this study, the promotion of AF depolymerization contributed to up-regulation of *OsVPE3* gene transcription and VPE activity, and accelerated PCD in aleurone layers, whereas the inhibitor Ac-YVAD-CMK reversed the effects of the AF depolymerizer. Therefore, due to inhibition of the depolymerization of AF, the AF stabilizer phalloidin not only reduced the levels of *OsVPE3* gene expression and VPE activity, but also delayed the process of PCD in rice aleurone layers. Similarly, the study also confirmed that the AF stabilizer, jasplakinolide, alleviated the occurrence of PCD in self-compatible pollen of *Papaver rhoeas* (Thomas et al., 2006). Moreover, the depolymerization inhibitor bistheonellide A increased elicitor-induced hypersensitive cell death by destroying the bundles of AF, showing that the depolymerization of AF plays an important role in the regulation of PCD in tobacco BY-2 cells (Higaki et al., 2007). We also found that Ac-YVAD-CMK attenuated the effect of the AF stabilizer phalloidin on OsVPE3, leading to prolonged PCD in the aleurone layers. Thus, it is confirmed that AF regulates OsVPE3-mediated PCD occurrence in aleurone layers.

However, the inhibitor Ac-YVAD-CMK alone significantly reduced *OsVPE3* mRNA transcription and VPE activity and decelerated the occurrence of PCD in aleurone layers. This result is supported by studies showing that the activation of caspase-1-like activity sped up the PCD process in plants (Vacca et al., 2006; Han et al., 2012; Ye et al., 2013). In addition to the effects of the inhibitor Ac-YVAD-CMK on the structure of AF, we also found that the vacuoles in the cells of aleurone layers treated with the inhibitor were smaller compared to cells treated with distilled water (Fig. 6). This suggests that the fusion of vacuoles is blocked, which means that the depolymerization of AF is hindered. Therefore, we deduced that OsVPE3 regulates vacuole fusion by mediating the change of AF structure. A previous study provided evidence that at the initial stage of leaf formation, the caspase-1 activity of leaves increases, followed by formation of the bundle of AF which then collapses, while the caspase-1 specific inhibitor prevents the change of AF structure (Lord et al., 2013). This agrees with the results obtained in this study. However, this also presents further evidence that the AF depolymerizer does not affect the activity of caspase-1 (Lord et al., 2013), which is inconsistent with the results of this study. Nonetheless, our results support the conclusion that the AF depolymerizer significantly up-regulated the levels of *OsVPE3* mRNA transcription and VPE activity in rice aleurone layers (Fig. 4). Furthermore, other studies have revealed that the AF depolymerizer Latrunculin (B) triggers the activity of caspase-3-like proteases in *Papaver rhoeas* pollen (Thomas et al., 2006), and the dynamic change of AF affects the caspase-3-like activity of Jurkat T cells (Posey and Biere, 1999; Odaka et al., 2000), showing that AF regulates caspase-like activities in plants.

## Conclusion

Unfortunately, there are few reports concerning the relationship between AF and VPE or the proteases with caspase-like activity in plants. However, based on the above evidence, we conclude that both the activation of VPE and the depolymerization of AF accelerate the process of vacuole fusion and PCD in rice aleurone layers, and there is an interaction between OsVPE3 and AF. Furthermore, the up-regulation of *OsVPE3* gene expression and VPE activity induced by endogenous GA and the dynamic change of AF structure are strongly related to the initiation and execution of PCD in rice aleurone layers. Further studies are needed to comprehensively elucidate the mechanism of AF and OsVPE3 or the proteases with caspase-like activity in regulating PCD in rice aleurone layers.

## Methods

### Plant Material and Growth Conditions

Rice (*Oryza sativa* L.) seeds were purchased from Hainan Danzhou seed company, China. Seeds were surface-sterilized with a solution of 0.1% potassium for 10 min, and then rinse with distilled water at least three times. Then, two methods were used to obtain the aleurone layers. One was that the aleurone layers were stripped from the intact seeds and de-embryo seeds after they were imbibed in distilled water for 4, 8, 12, 16, and 18 h. The other was that the aleurone layers were isolated from the intact seeds by removing the starch endosperm after 12 h of imbibition, then the isolated layers were incubated in 10 *μ*M Ac-YVAD-CMK (dissolved in DMSO), 10 *μ*M Ac-DEVD-CHO (dissolved in DMSO), 10 *μ*g/mL phalloidin (dissolved in DMSO), and 10 *μ*g/mL cytochalasin B (dissolved in DMSO); the layers incubated in distilled water were regarded as the control (Con). According to the different experimental requirements, the separated aleurone layers were cultured in different treatment solutions and cultured at 27^∘^C in an incubator, the micro-observation or corresponding indices were deleted at different times, and each treatment was repeated at least three times.

### Cell Viability Assay

Cell viability was assayed according to the method of Wu et al. (Wu et al., 2016) with some modifications. The layers were soaked in 20 mM CaCl _2_ for 30 min, stained with 2 *μ*g/mL fluorescein diacetate (FDA) in 20 mM CaCl _2_ for 30 min and subsequently washed with 20 mM CaCl _2_ for 10 min. After the drug was washed out, a staining solution containing 2 *μg*/mL-1 N-(3-triethylammoniumpropyl)-4-(6-(4-(diethylamino) phenyl)-hexatrienyl) pyridinium dibromide (FM4-64) in 20 mM CaCl _2_ was used for 20 min, followed by 20 mM CaCl _2_ for 10 min to wash away the excess FDA. The stained layers were observed, and the images were acquired under a laser scanning confocal microscope (LSCM; Olympus, Fluoview 1000). The FV10-ASW 1.6 Viewer software was used with the following parameters: green excitation wavelength at 488 nm; orange excitation wavelength at 568 nm; power 5%; and medium scan. The number of live and dead cells in at least four different fields in a sample was counted to determine the proportion of viable cells.

### VPE Activity Assay

VPE activity was measured using the method reported by Kuroyanagi et al. and Wang et al. (Wang et al., 2009; Kuroyanagi et al., 2005) with some modifications, and quantified using the Bradford method (Bradford 1976). The isolated aleurone layers were homogenized in an extraction buffer of 100 mM NaAc (pH 5.5), 100 mM NaCl, 1 mM EDTA, and 100 mM DTT under ice-cold conditions. The homogenate was centrifuged at 15,000 g for 20 min at 4^∘^C, and the supernatant was pre-incubated in 100 mM NaAc and 100 mM DTT before adding the substrate, Ac-ESEN-MCA (Ac-Glu-Ser-Glu-Asn-MCA) (10 mM). The mixtures were incubated for 2 h at 20^∘^C, then the fluorescence intensity values were monitored at an excitation wavelength of 360-380 nm and an emission wavelength of 460-480 nm using a fluorescence microplate reader (Synergy HTX, BioTek, Vermont, USA).

### Caspase Activity Assay

Caspase-1 and caspase-3 activity was analyzed according to the method of Li et al. (2011) and quantified using the Bradford method (Bradford 1976). Seventy-five pieces of layers were harvested and homogenized in an enzyme lysate under ice-cold conditions. The homogenate was centrifuged at 15,000 g for 15 min at 4^∘^C, and the supernatant was used to measure caspase-1 and caspase-3 activity. Acetyl-Tyr-Val-Ala-Asp p-nitroanilide (Ac-YVAD-pNA) and Acetyl-Asp-Glu-Val-Asp p-nitroanilide (Ac-DEVD-pNA) were the substrates of caspase-1 and caspase-3, respectively. Caspase-1 activity and caspase-3 activity were detected using kits according to the manufacturer’s instructions (Beyotime Institute of Biotechnology, Beijing, China). The standard activity curves of caspase-1 and caspase-3 were determined by their ability to change Ac-YVAD-pNA and Ac-DEVD-pNA into the yellow formazan product pNA. The detection buffer and the substrates of caspase-1 and caspase-3 were added and the absorbance was determined with a fluorescence microplate reader (Synergy HTX, BioTek, Vermont, USA) at 405 nm.

### Actin Microfilament Morphology Observation

The experiment was performed using the method of Adams and Pringle (Adams and Pringle, 1991) with minor modifications. The aleurone layers were immersed in 20 mM CaCl _2_ solution for 30 min, then fixed with 4% polyoxymethylene solution and incubated at room temperature for 30 min. After the completion of incubation, the layers were washed with PBS buffer three times, permeated three times every 5 min in 0.1% Triton X-100 (in PBS buffer), and rinsed with PBS buffer three times. The fixed aleurone cells were placed in 96-well enzyme plates containing 100 *μ*L FITC-labeled phalloidin (resolved in 1% BSA) and stained for 60 min, and then the stained cells were gently washed with PBS buffer three times. The stained cells were observed and imaged under a LSCM (Fluoview 1000, Olympus). The excitation wavelengths of FITC were selected, at medium speed scanning and 5% power, and the images were analyzed by FV10-ASW1.6 Viewer software.

### Quantitative Real-time Fluorescence PCR

Total RNA was isolated from the whole seeds, aleurone layers, embryos, and starch endosperm treated with different treatments using TRIzol Reagent (Tiangen, Beijing, China) according to the manufacturer’s instructions. The concentration of RNA was quantified using an ELISA instrument (Synergy, HTX, BioTek). Reverse transcription was performed using the PrimeScript RT reagent Kit (TaKaRa, Dalian, China), following the manufacturer’s procedures. For the first-strand, cDNA was synthesized from 2 *μ*g of total RNA with a ReverTra Ace qPCR kit (Toyobo). Quantitative Real-Time Fluorescence PCR (qRT-PCR) was performed using the SYBR Green real-time PCR Master Mix (TianGen, Beijing, China), following the manufacturer’s instructions. The primers were as follows: *OsVPE1* (forward, 5’-GTGCCATGCGTACCAGAT-3’, and reverse, 5’-TAACCTTCCCACTCCCTC-3’) (accession no. BAF15342); *OsVPE2* (forward, 5’-CGGCTCCAACGGCTACTACAAC-3’, and reverse, 5’-TCGGGACCCCAGCATAGACA-3’) (accession no. BAF05258); *OsVPE3* (5’-CGGTAACTACAGGCACC AGGC-3’, and reverse, 5’-GTGACTTCGTCTCCAG TCTAATCC-3’) (accession no. BAC41387); *OsVPE4* (forward, 5’-CAAAGGCAGCCACTCCTACAC, and reverse, 5’-GCACTCCCAGTCCTCAACCAG-3’) (accession no. BAF18418); OsActin (forward, 5’-TCTCTC TGTATGCCAGTGGTCGT-3’, and reverse, 5’-TCAT AGTCCAGGGCGATGTAGG-3’) (accession no. NM_ 001057621). OsActin was used as a reference.

### Statistical Analysis

The data reflect the means ±*SE* of at least three individual experiments, and Duncan’s multiple range test was used to detect statistically significant differences (*P*<0.05).

## Abbreviations

Ac-DEVD-pNA: Acetyl-Asp-Glu-Val-Asp p-nitroanilide
Ac-ESEN-MCA: Ac-Glu-Ser-Glu-Asn-MCA
Ac-YVAD-pNA: Acetyl-Tyr-Val-Ala-Asp p-nitroanilide
AFs: Actin filaments
Asp: Aspartic acid
CB: Cytochalasin B
CD: Cytochalasin D
F-actin: Fibrous actin
FDA: Fluorescein diacetate
FM4-64: 1N-(3-triethylammoniumpr- opyl)-4-(6-(4-(diethylamino) phenyl)-hexatrienyl) pyridinium dibromide
G-actin: Spherical actin
LSCM: Laser scanning confocal microscopy
MFs: Actin microfilaments
PCD: Programmed cell death
qRT-PCR: Quantitative real-time fluorescence PCR
VPE: Vacuolar processing enzyme
